# Progress and Prospect of Immunotherapy for Triple-Negative Breast Cancer

**DOI:** 10.3389/fonc.2022.919072

**Published:** 2022-06-20

**Authors:** Chenyi Luo, Peipei Wang, Siqi He, Jingjing Zhu, Yuanyuan Shi, Jianxun Wang

**Affiliations:** ^1^ School of Life Sciences, Beijing University of Chinese Medicine, Beijing, China; ^2^ Shenzhen Research Institute of Beijing University of Chinese Medicine, Shenzhen, China

**Keywords:** immunotherapy, antibody–drug conjugate, bispecific antibody, immune checkpoint inhibitor, neoantigen cancer vaccine, oncolytic virus, tumor -infiltrating lymphocyte, chimeric antigen receptor T cell

## Abstract

Breast cancer is the most commonly diagnosed cancer (estimated 2.3 million new cases in 2020) and the leading cause of cancer death (estimated 685,000 deaths in 2020) in women globally. Breast cancers have been categorized into four major molecular subtypes based on the immunohistochemistry (IHC) expression of classic hormone and growth factor receptors including the estrogen receptor (ER), progesterone receptor (PR), and human epidermal growth factor receptor 2 (HER2), as well as a proliferation marker Ki-67 protein expression. Triple-negative breast cancer (TNBC), a breast cancer subtype lacking ER, PR, and HER2 expression, is associated with a high metastatic potential and poor prognosis. TNBC accounts for approximately only 15%–20% of new breast cancer diagnoses; it is responsible for most breast cancer–related deaths due to the lack of targeted treatment options for this patient population, and currently, systemic chemotherapy, radiation, and surgical excision remain the major treatment modalities for these patients with TNBC. Although breast cancer patients in general do not have a robust response to the immunotherapy, a subset of TNBC has been demonstrated to have high tumor mutation burden and high tumor-infiltrating lymphocytes, resembling the features observed on melanoma or lung cancers, which can benefit from the treatment of immune checkpoint inhibitors (ICIs). Therefore, the immunogenic nature of this aggressive disease has presented an opportunity for the development of TNBC-targeting immunotherapies. The recent US Food and Drug Administration approval of atezolizumab in combination with the chemotherapeutic agent nab-paclitaxel for the treatment of PD-L1-positive unresectable, locally advanced, or metastatic TNBC has led to a new era of immunotherapy in TNBC treatment. In addition, immunotherapy becomes an active research area, both in the cancer biology field and in the oncology field. In this review, we will extend our coverage on recent discoveries in preclinical research and early results in clinical trials from immune molecule-based therapy including cytokines, monoclonal antibodies, antibody–drug conjugates, bi-specific or tri-specific antibodies, ICIs, and neoantigen cancer vaccines; oncolytic virus-based therapies and adoptive immune cell transfer–based therapies including TIL, chimeric antigen receptor-T (CAR-T), CAR-NK, CAR-M, and T-cell receptor-T. In the end, we will list a series of the challenges and opportunities in immunotherapy prospectively and reveal novel technologies such as high-throughput single-cell sequencing and CRISPR gene editing-based screening to generate new knowledges of immunotherapy.

## Introduction

The very beginning of immunotherapy might be traced back to the China’s Qin dynasty period, around the third century BC (1–6). William Bradley Coley, who is known today as the Father of Immunotherapy, first attempted to develop immune-based treatment for cancer by injecting different mixtures of live and inactivated *Streptococcus pyogenes* and *Serratia marcescens* into patients’ tumors in 1891 (7). However, the curative power of immunotherapy for cancer treatment is well demonstrated until recently. The US Food and Drug Administration (FDA) approved Bacille Calmette-Guérin (BCG) in 1990, a bacterial vaccine against tuberculosis, to treat early-stage bladder cancer, the first FDA-approved immunotherapy. The approval of immune checkpoint inhibitors (ICIs) Yervoy (ipilimumab) in 2011, Keytruda (pembrolizumab) in 2014, and Opdivo (nivolumab) in 2015 by the US FDA are the major milestones of immunotherapy, followed by the approval of Kymriah (tisagenlecleucel) and Yescarta (axicabtagene ciloleucel) in 2017, and the most recent approval of Kimmtrak (tebentafusp) in 2022 adopted cell transfer–based CAR-T and TCR-T therapies. Now, immunotherapy is considered to be the fifth pillar of cancer treatment along with surgery, chemotherapy, radiotherapy, and target therapy.

Breast cancer remains the most diagnosed cancer in women globally. It is estimated to have approximately 281,550 new cases and 43,600 deaths in 2021 in the United States (8). In addition, a recent nationwide statistics report estimated 306,000 new cases and 71,700 deaths in 2016 in China (9). Breast cancers are traditionally categorized into four molecular subtypes based on the IHC expression of classic hormone and growth factor receptors including the estrogen receptor (ER), progesterone receptor (PR), and human epidermal growth factor receptor 2 (HER2), as well as a proliferation marker Ki-67 protein expression (2). The 2013 St. Gallen International Breast Cancer Conference released a new definition of breast cancer molecular subtypes: luminal A (ER/PR^+^, HER2^−^, Ki67^+^ < 20%, with the percentage indicating the IHC staining results for patient samples), luminal B (ER/PR^+^ < 20%, HER2^−^, Ki67^+^ ≥ 20%); HER2^+^ B2 (ER/PR^+^, HER2 overexpression), HER2 overexpression (ER^−^, PR^−^, HER2 overexpression), basal-like triple-negative breast cancer (TNBC, ER^−^, PR^−^, and HER2^−^), and other special subtypes (10). Breast cancer patients with luminal A or luminal B subtypes can be treated with endocrine therapies including selective estrogen receptor modulators, aromatase inhibitors, and ER degraders (3–5, 11–13). Patients with HER2 overexpression are candidates for receiving HER2-targeting monoclonal antibodies (mAbs), antibody–drug conjugates (ADCs), or tyrosine kinase inhibitors (3–5, 11–13). While these three subtypes above can have favorable clinical outcomes due to their responsiveness to the targeted therapies, poor prognosis is usually observed within a major subdivision of the fourth breast cancer subtype referred to as TNBC with a negative expression of ER, PR, or HER2 due to lack of targeted treatment options for this patient population (3–5, 11–13). Although breast cancer patients in general do not have a robust response to the immunotherapy, a subset of TNBC has been demonstrated to have high tumor mutation burden (TMB) and high tumor-infiltrating lymphocytes (TILs), resembling the features observed on melanoma or lung cancers that can benefit from the treatment of ICIs. Therefore, the immunogenic nature of this aggressive disease has presented an opportunity for the development of TNBC-targeting immunotherapies (11–13).

The recent FDA approval of atezolizumab in combination with the chemotherapeutic agent nab-paclitaxel for the treatment of PD-L1-positive unresectable, locally advanced, or metastatic TNBC has led to a new era of immunotherapy in TNBC. The final market approval of the combination therapy was based on the encouraging results from the Impassion130 clinical trial, with a demonstration of the median overall survival (OS) for atezolizumab/nab-paclitaxel-treated patients with PD-L1-positive TNBC extended nearly 10 months in comparison with patients treated with placebo/nab-paclitaxel (NCT02425891).

Immunotherapy has become an active research area, both in the cancer biology field and in the oncology field. In this review, we will extend our coverage on recent discoveries in preclinical research and results in clinical trials from immune molecule-based therapy including cytokines, mAbs, ADCs, bi-specific or tri-specific antibodies, ICIs, and neoantigen cancer vaccines; oncolytic virus-based therapies and adoptive immune cell transfer–based therapies including TIL, chimeric antigen receptor-T (CAR-T), CAR-NK, CAR-M, and T-cell receptor-T (TCR-T). In the end, we will list a series of the challenges and opportunities in immunotherapy prospectively and reveal novel technologies such as high throughput single-cell sequencing and CRISPR gene editing-based screening to generate new knowledges of immunotherapy.

## Immune Molecule-Based Therapy

### Cytokine

Cytokines are major regulators of the innate and adaptive immune systems that control the proliferation, differentiation, survival, and effector functions of leukocytes through communication over short distances in paracrine and autocrine fashion in immune systems (14), with a potential to enhance the anti-tumor immune response. Since the discovery of interleukin-1 (IL-1) as an “endogenous pyrogen” in 1953, the use of exogenous cytokines for cancer treatment by manipulating a patient’s own immune system has been actively pursued in basic and clinical research (15). Currently, two cytokines have been approved by the FDA for cancer therapy (16). Interferon alpha 2 (IFN-α) is the first cytokine to win FDA approval as a single-agent cytokine therapy for cancer in 1986. After Rosenberg’s seminal discovery showing that injections with T-cell growth factor later named interleukin-2 (IL-2) can shrink tumors in humans (17), IL-2 was approved by the FDA as an immunotherapeutic cytokine monotherapy for the treatment of metastatic kidney cancer in 1991 and later for metastatic melanoma in 1998.

Aside from IFN-α and IL-2, other cytokines such as the tumor necrosis factor (TNF), interleukin-12 (IL-12), interleukin-15 (IL-15), interleukin-21 (IL-21), and granulocyte-macrophage colony stimulating factor (GM-CSF) have been undergoing the safety and efficacy evaluation for cancer treatments in multiple clinical trials. In addition, currently, more than 40 identified cytokines are approved as single-agent immunotherapies for a limited number of indications other than cancer treatment.

TNF was discovered as the first immune molecule to have robust activity to kill cancer cells in 1975. However, the initial clinical trials with TNF treatment encountered the high systemic toxicity. With the development of novel TNF administration procedures, such as high concentrations of TNF perfusion in isolated limbs of patients with melanoma or sarcoma, the therapeutic efficacy of TNF has been demonstrated in clinical trials. Nevertheless, physiological intra-tumor TNF levels are likely insufficient to induce cancer regression in patients; the TNF dosage to have a robust tumor-killing effect without a severe side effect is still a big challenge to reach in clinical trials, and many studies are currently undergoing (18, 19).

IL-12 was discovered as a potent, pro-inflammatory type I cytokine with a potential for cancer treatment as an immunotherapy in 1989. Similar to TNF, early clinical trials found dose-limiting toxicities with a systemic delivery of IL-12. The invention of novel delivery systems may lead to fulfill the potential of IL-12 as a potent anti-tumor cytokine in the near future (20).

Both IL-15 and IL-21 are members of the IL-2 family and have been investigated to evaluate their therapeutic potentials for cancer treatment (21, 22).

GM-CSF, discovered as a potent cytokine promoting the differentiation of myeloid cells in 1993, is currently undergoing investigation as an adjuvant immunomodulator agent to elicit anti-tumor immunity in basic and clinical research (23).

Although cytokines as monotherapy agent for cancer treatment have many advantages such as manufacture and administration, most cytokines have failed in clinical trials as monotherapy for many reasons including insufficient cytokine concentrations in the tumor when cytokine is administered parenterally, severe toxicities associated with cytokine administration and induction of humoral or cellular checkpoints (14). Many strategies have been investigated clinically to circumvent the impediments encountered during cytokine administration, such as cytokines in combination therapy with checkpoint inhibitors, cytokines in combination therapy with anticancer mAbs to increase the antibody-dependent cellular cytotoxicity (ADCC) of these antibodies, and antibody–cytokine fusion proteins to facilitate tumor-specific immune responses. At the same time, cytokines in combination therapy with oncolytic virus have been investigated clinically. Talimogene laherparepvec (T-VEC; Imlygic™) is an oncolytic herpes simplex virus that uses GM-CSF expression as an immune enhancer and has gained FDA approval for cancer immunotherapy in 2015 (T-VEC will be covered more in the next section), indicating cytokines can enhance the oncolytic virus-induced immune response against tumors.

### Monoclonal Antibody and Antibody–Drug Conjugate

mAbs or ADCs can recognize tumor-specific or tumor-overexpressed antigens and kill tumor cells through antigen-dependent cell-mediated cytotoxicity (ADCC) or inhibit tumor growth by the drug conjugated on the antibody. Trastuzumab, also known as Herceptin, Ogivri, or Herzuma, is an mAb targeting epidermal growth factor receptor 2 (HER2), the first recombinant antibody to be commercially approved as cancer drug by the FDA in 1997. Because TNBC patients are HER2 negative and not suitable for Trastuzumab or other therapies targeting HER2, novel tumor cell–specific markers such as vascular endothelial growth factor (VEGF) A, cathepsin D (cath-D) and CD40 are identified and mAb-based targeted therapy appro?A3B2 ?>aches are undergoing for TNBC treatment.

Bevacizumab, also known as Avastin is a humanized mAb targeting VEGF-A, as one of the first targeted therapies and the first angiogenesis inhibitor approved by the FDA in 2009. Initially approved for the treatment of metastatic colorectal cancer in combination with chemotherapy, its indications have been extended to metastatic breast cancer, non-small-cell lung cancer, glioblastoma, renal cell carcinoma, ovarian cancer, and cervical cancer (24). In addition to the major role of VEGF in controlling blood vessel formation, it is now known that it also modulates tumor-induced immunosuppression. Therefore, the immunomodulatory properties of bevacizumab have been investigated as new perspectives for combination therapy approaches in clinical trials.

Cath-D is an aspartic protease and a tumor-specific extracellular target in TNBC. An immunomodulatory antibody-based strategy against cath-D is a promising immunotherapy currently in the developmental stage to treat patients with TNBC (25).

CD40 is a member of the TNF receptor superfamily and licenses dendritic cells to promote anti-tumor T-cell activation and re-educate macrophages to destroy tumor stroma upon activation. CD40 antibodies with agonist activity have been developed and evaluated in clinic trials (26), and major tumor regressions have been observed in patients with breast cancer, pancreatic cancer, mesothelioma, and other tumors in combination with chemotherapy when CD40 antibodies are used with and without anti-CTLA4 mAb) therapy.

ADCs represent an interesting new class of anticancer agents, utilizing the specificity of mAbs on cellular-antigen recognition for a targeted release of potent cytotoxic drugs, with a potentially increased activity and reduced toxicity compared with traditional chemotherapies (27, 28).

Trastuzumab deruxtecan (DS-8201) is an ADC composed of an anti-HER2 antibody coupled to a cytotoxic topoisomerase I inhibitor by a cleavable tetrapeptide-based linker. It has recently received the approval of the FDA to treat trastuzumab emtansine (TDM1)-pretreated patients with breast cancer, possibly because of its ability to exert cytotoxic activity against antigen-negative cells, a so-called bystander effect (29). It has been recently reported that Trastuzumab in combination with chemotherapy can be considered as a new standard option for patients with HER2-positive advanced gastric or gastro-esophageal junction cancer after the evaluation of a Toga phase III trial (30), suggesting Trastuzumab in combination with chemotherapy should be evaluated for TNBC treatment in the clinical trial. In addition, disitamab vedotin, a novel ADC comprising a HER2 mAb conjugated *via* a cleavable linker to the cytotoxic agent monomethyl auristatin E, received its first Biologics License Application (BLA) approval in China in 2021 for the treatment of patients with HER2-overexpressing locally advanced or metastatic gastric cancer (31). In addition, disitamab vedotin as monotherapy or combination therapy is also in clinical trials for the treatment of other solid tumors, including urothelial cancer, biliary tract cancer, non-small cell lung cancer, and HER2-positive and HER2-low-expressing breast cancers.

Sacituzumab govitecan is an ADC composed of an antibody targeting the human trophoblast cell-surface antigen 2 (Trop-2), coupled to SN-38 (topoisomerase I inhibitor) through a proprietary hydrolyzable linker for the treatment of triple-negative breast tumors. In a recent clinical trial, it has been reported that sacituzumab govitecan-hziy was associated with durable objective responses in patients with heavily pretreated metastatic TNBC (32). In another clinical trial, it has been reported that progression-free survival and OS were significantly longer with sacituzumab govitecan than with single-agent chemotherapy among patients with metastatic TNBC (33).

In addition to HER2 and Trop2, other ADC targets including zinc transporter LIV-1 (solute carrier family 39 member 6, SLC39A6) and folate receptor alpha (FRα) have also been investigated for TNBC treatment in clinical trials (27, 28).

The identification of TNBC-specific antigens such as Trop2 and LIV-1 could lead to an efficient target therapy approach to treat TNBC, similar to Her2 mAb for Her2^+^ breast cancer treatment.

### Bispecific Antibody and Multi-Specific Antibody

A bispecific antibody is a kind of bi-functional protein composed of two different fragments and specifically binds to two different types of antigen, to connect tumor cells with immune cells (T- or NK-engager) or inhibit two signaling pathways synergistically and simultaneously (such as EGFR-Notch bsAb).

Blinatumomab, also known as Blincyto, is a CD3 × CD19 bispecific T-cell engager (BiTE) and won the approval of the FDA in 2014, for the treatment of acute lymphoblastic leukemia (pre-B-ALL), marking the milestones of the therapeutic utility of the bispecific antibody (bsAb) in cancer immunotherapy. In addition, the bispecific antibody-based therapeutics for the treatment of TNBC have also gained more attention in the scientific community recently (34).

**Figure 1:**
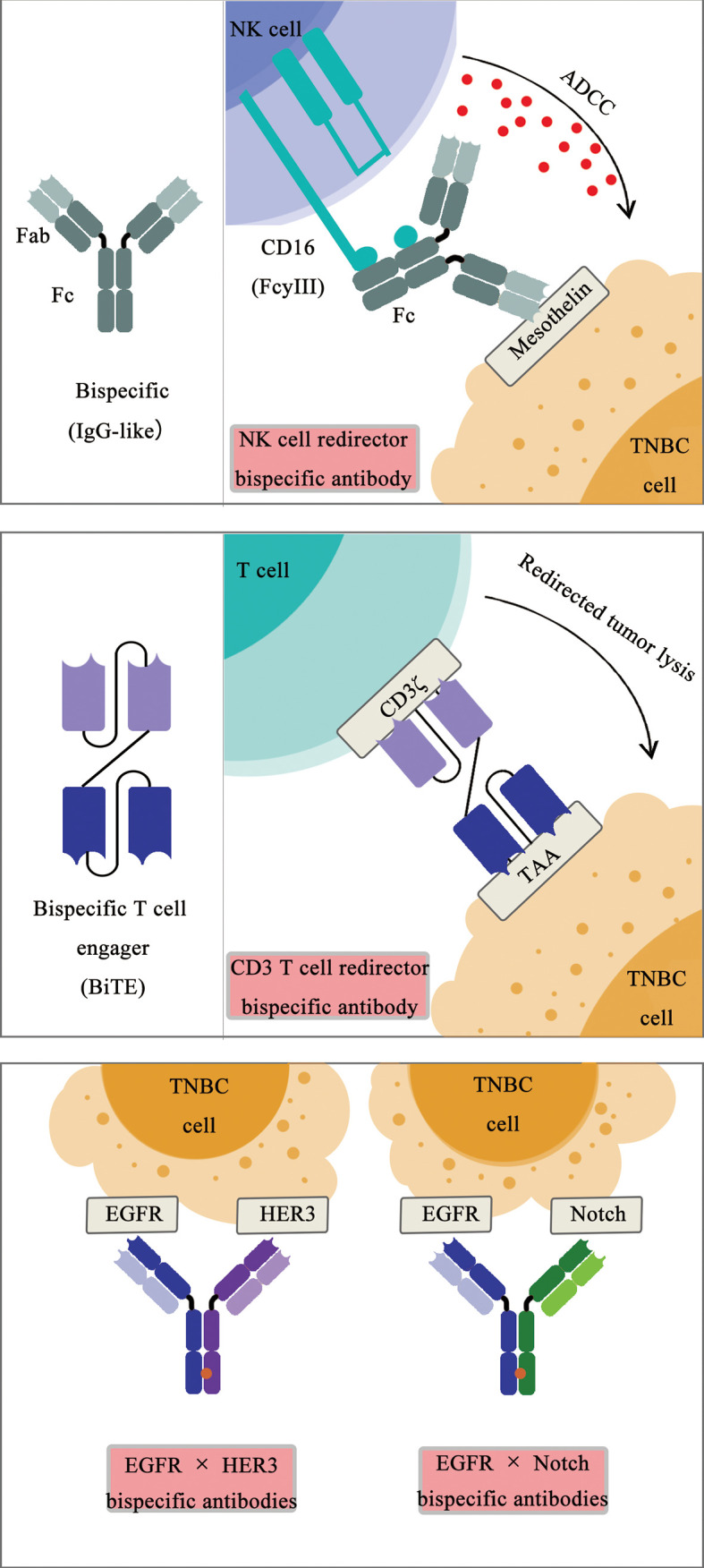
Schematic diagram of bispecific antibodies.

Among many bispecific antibodies under development for the treatment of TNBC, most bsAbs are categorized as CD3+ T-cell engagers (35, 36), targeting Trop2, carcinoembryonic antigen–related cell adhesion molecule 5 (CEACAM5), ephrin receptors A10 (EphA10), P-cadherin, epithelial cell adhesion molecule (EpCAM), EGFR, and mesothelin using different recombinant protein engineering design strategies (**Figure 1**). The CD3 × Trop2 and CD3 × CEACAM5 bsAbs were recently generated using the DOCK-AND-LOCK (DNL) technology platform (37). The CD3 × EphA10 bsAb was generated as a diabody by fusing scFv fragment A (VL chain of EphA10 linked to the VH chain of CD3) to scFv fragment B (VL chain of CD3 linked to the VH chain of EphA10) (38). The CD3 × P-cadherin bsAb (PF-06671008) was generated using a bsAb platform known as the dual-affinity retargeting (DART) scaffold (39). Catumaxomab, a CD3 x EpCAM bsAb, was generated to target chemo-resistant EpCAM-positive TNBC cells (40). The CD3 × EGFR BiTE was generated, and its cytotoxic activity against EGFR-expressing TNBC cells was enhanced by blocking the immune checkpoint receptor T-cell immunoreceptor with immunoglobulin and ITIM domains (TIGIT) or its ligand poliovirus receptor (PVR) (41). Using CD3 and EGFR targeting antibodies with a different approach, the synthetic multivalent antibodies retargeted exosomes (SMART-Exos) nanomedicine platform was designed to redirect and activate cytotoxic T cells toward TNBC cells, inducing a potent anti-tumor immune response in a human TNBC xenograft mouse model (42). Similar to CD3^+^ T-cell engagers, a Fab-like CD16 (FcγRIII) × mesothelin bsAb was generated to recruit NK cells to infiltrate into mesothelin-expressing TNBC tumors, inducing a classic antibody-dependent cellular cytotoxicity (ADCC) mechanism upon the binding of CD16 on NK cells to the Fc region of the antibody bound to mesothelin on TNBC (43).

In addition to immune cell (T and NK) engagers, bsAb simultaneously targeting receptors on TNBC cells, including EGFR, HER3, and Notch, have been generated and under evaluation recently (**Figure 1**). An EGFR × human epidermal growth factor receptor 3 (HER3) bispecific diabody-Fc fusion protein was recently generated, inhibiting the proliferation of TNBC cells (44, 45). Similarly, an EGFR × Notch bsAb was generated, promoting the therapeutic response of TNBC cells to PI3K inhibition (46). A CD3 × MUC1 bsAb was generated to elevate the efficacy of an activated cytokine-induced killer (CIK) for the treatment of advanced breast cancer (47). An anti-TGFβ/PD-L1 bifunctional fusion protein and other therapeutics targeting TGFβ in TNBC are under investigation in clinical trials for TNBC treatment (48).

The identification of TNBC-specific antigens such as Trop2 and LIV-1 could also lead to an efficient immunotherapy approach to treat TNBC, similar to CD19-CD3 bsAb for pre-B ALL treatment.

### Immune Checkpoint Inhibitor

An ICI can unleash an immune system attack on cancer cells, which is usually suppressed by tumor cells or the tumor microenvironment. The FDA approval of the ICI anti CTLA-4 antibody Yervoy (ipilimumab) in 2011, anti-PD1 antibodies Keytruda (pembrolizumab) in 2014, and Opdivo (nivolumab) in 2015 are the major milestones of immunotherapy, marking a beginning of new era of cancer therapy (49). Later, the anti-PD1 antibody Libtayo (cemiplimab) and anti-PD-L1 antibodies Tecentriq (atezolizumab), Bavencio (avelumab), and Imfinzi (durvalumab) have been approved by the FDA (**Table 1**).

**Table 1:** Immune checkpoint inhibitor and its application on TNBC treatment: (A) Immune checkpoint inhibitors.

Target	Antibody	Trade name	Isotype	Initial approval time
CTLA-4	Ipilimumab Tremelimumab	Yervoy /	IgG1 IgG2	2011/325 2015/4/15
PD-1	Pembrolizumab Nivolumab Cemiplimab	Keytruda Opdivo Libtayo	IgG4 IgG4 IgG4	2014/9/5 2015/6/22 2021/2/22
PD-L1	Atezolizumab Avelumab Durvalumab	Tecentriq Bavencio Imfinzi	IgG1 IgG1 IgG1	2016/5/18 2017/3/23 2017/5/21

ICI therapy has been demonstrated to generate durable responses in a variety of tumors such as melanoma and lung cancer and become the most successful immune-based intervention for cancer therapy. mAbs against PD-1/PD-L1 and CTLA-4 have become powerful tools to release the inhibitory regulation of T-cell activation from tumor cells or the tumor microenvironment (TME) (49).

Breast cancer patients in general are not predicted to benefit from immunotherapy due to modest TMB (approximately 1 mutation/Mb) compared to melanoma or other tumors with high TMB. However, a subset of TNBC has been demonstrated to have high TMB (>10 mutations/Mb) and high TILs, resembling to features observed on melanoma or lung cancers which can benefit from ICI treatment. Therefore, the immunogenic nature of this aggressive disease has presented opportunity for the development of TNBC-targeting immunotherapies (49–51).

The heterogeneity feature of TNBC suggests that only a subpopulation of patients could benefit from ICI treatment. Therefore, it is a challenge to select patients that will be predicted to benefit the most. A few parameters have been considered to evaluate the potential of immunotherapy in TNBC or other types of breast cancer, such as TMB and neoantigen load, the diversity of the immune infiltrate, and the gut and breast microbiomes. Since low TMB is associated with poor prognosis, only a fraction of patients with high TMB could have benefits from ICI treatment. In addition, micro-satellite instability or BRAC1/2, PTEN mutations also have predictive values for patients’ potential benefit from ICI treatment. The number of TILs is another predictive marker; a high number of TILs is generally associated with better prognosis. PD-L1 expression in tumor is also an important marker to predict patients’ outcome with ICI treatment. PD-L1 expression is currently conducted using five distinct FDA-approved companion diagnostic immunohistochemistry tests for the routine clinical test (52). Since soluble PD-L1 (sPD-L1) has been detected in the peripheral blood of many cancer patients including advanced non-small cell lung cancer, multiple myeloma, diffuse large B-cell lymphoma, and renal cell carcinoma, and high levels of sPD-L1 are associated with poor prognosis, sPD-L1 can serve as a surrogate marker for PD-L1 on TNBC. Furthermore, it has become a consensus that a combination of predictive biomarkers such as PD-L1 expression, intratumoral TIL (iTIL) and stromal TIL (sTIL) density together with TMB, TCR diversity, and immune gene signatures will more likely yield an improved performance over each of these biomarkers alone and warrant further investigation (49).

Many clinical trials with ICI therapies for TNBC are completed or still undergoing; to summarize the current status of clinical results, the response rates of single-agent ICIs in mTNBC may be modest. However, the durable responses of a subset of PD-L1 positive patients suggest that the combination treatment of immune checkpoint blockade with other treatment modalities may provide a favorable outcome (**Table 1**).

**Table 1:** (B) Clinical trials using PD1/PD-L1 antibodies in TNBC.

	Treatment	Clinical trial ID	Intervention	Phase Trial stage	Related results	Disease setting
Monotherapy	PD1/PD-L1antibody	NCT01848834 NCT02447003 NCT02555657 NCT02981303 NCT03197389	Pembrolizumab Pembrolizumab Pembrolizumab vs chemotherapy Pembrolizumab Pembrolizumab	I Completed II Completed III Active II Completed I Completed	ORR 18. 5 % ORR 5.7%;PFS 2.0mths; OS 9.0 mths, fifirst line setting:ORR 21.4%; PFS 2.1 mths; OS 18.0 mths No difference in PFS and OS No Results Posted Not Specified	recurrent or metasttatic metasttatic metasttatic metasttatic Early stage
		NCT01375842 NCT03281954 NCT01772004 NCT02926196 NCT02489448	Atezolizumab Atezolizumab Avelumab Avelumab Durvalumab	I Completed III Recruiting I Completed III active I/II Completed	ORR 6% (12 vs 0%*): OS (10.1 vs 6.0 mths*), fifirst line setting:ORR 24%; OS 17.6 mths; ORR 5.2%(22.2 vs 2.6**) / Not Specified / No Results Posted	metasttatic recurrent or metastatic recurrent or metastatic recurrent or metastatic recurrent or metastatic
	PD1/PD-L1 antibody + Chemotherapy	NCT02819518 NCT02755272 NCT02513472 NCT01042379 NCT02622074 NCT03036488 NCT02499367	Pembrolizumab + Nab-paclitaxel or Paclitaxel or Gemcitabine/Carboplatin Pembrolizumab + Gemcitabine/Carboplatin Pembrolizumab + Eribulin mesylate neoadjuvant Pembrolizumab + Paclitaxel, followed by AC, neoadjuvant Pembrolizumab + cxhemotherapy combination, (Nab-paclitaxel, Paclitaxel, Doxorubicin, Cyclophosphamide, Carboplatin) neoadjuvant Pembrolizumab + Paclitaxel-Carboplatin followed by adjuvant Pembrolizumab Cyclophosphamide, Cisplatin or Doxorubicin followed by Nivolumab	III Active II Recruiting I/II Completed II Recruiting I Completed III Active II Active	first line setting: PFS 9.7 mths / ORR 25.6%; PFS 4.1 mths / first line setting: pCR 60% first line setting: pCR 64.8% ORR 35 % (doxorubicin), first line setting: ORR 17%	locally advanced, metastatic metastatic metastatic locally advanced, metastatic locally advanced locally advanced metastatic
		NCT01633970 NCT02425891 NCT03125902 NCT03371017 NCT02620280 NCT03197835 NCT03281954 NCT03498716 NCT03164993 NCT00856492 NCT02685059	Atezolizumab + Nab-paclitaxel Atezolizumab + Nab-paclitaxel Atezolizumab + Paclitaxel Atezolizumab + Gemcitabine/Carboplatin or Capecitabine Neoadjuvant Atezolizumab + Nab-paclitaxel + Carboplatin, followed by AC or EC or FEC Neoadjuvant Atezolizumab + Nab-paclitaxel, followed by AC Neoadjuvant Atezolizumab + Paclitaxel + Carboplatin, followed by adjuvant Atezolizumab + Paclitaxel, followed by Atezolizumab + AC or EC Atezolizumab + Pegylated liposomal doxorubicin + Cyclophosphamide Durvalumab + Cyclophosphamide + Doxorubicin hydrochloride + Paclitaxe Neoadjuvant Durvalumab + Nab paclitaxel + EC	I Completed III Completed III Active III Recruiting III Active III Active III Recruiting III Recruiting II Recruiting II Completed II Completed	ORR 39.4%:PFS 5.5 mths First line setting: OR 53%; OS 25 mths / / / pCR 57.6% / / / Not Specefied pCR 53%	locally advanced, metastatic locally advanced, metastatic locally advanced, metastatic locally advanced, metastatic larly high risk, locally advance early stage early stage locally advanced locally advanced locally advanced early stage
Combination	PD1/PD-L1 antibody + Targeted therapy	NCT02657889 NCT04683679 NCT03106415 NCT03797326 NCT02834247 NCT02849496 NCT02322814 NCT03971409 NCT03167619 NCT03801369 NCT02484404 NCT02734004	Pembrolizumab + Niraparib Pembrolizumab + Olaparib Pembrolizumab + MEK_i_ Pembrolizumab + Lenvatinib Nivolumab + TAK-659 Atezolizumab + Olaparib Atezolizumab + Taxanes + MEK_i_ Avelumab + MEK_i_ Durvalumab + Olaparib Durvalumab + Olaparib Durvalumab + Olaparib +/-VEGFR_i_ Durvalumab + Olaparib +/-VEGFR	I/II Completed II Recruiting I/II Active II Active I Completed II Suspended II Completed II Recruiting II Active II Recruiting I/II Recruiting I/II Completed	ORR 29% (67% #) / / / Not Specified / ORR 29-34% / / / / Not Specified	locally advanced or metastatic metastatic or recurrent locally advanced or metastatic metastatic metastatic locally advanced or metastatic metastatic or locally advanced metastatic or locally advanced locally advanced or metastatic metastatic metastatic or recurrent advance or metastatic
	PD1/PD-L1 antibody + NK cell	NCT04551885 NCT03387085	Avelumab + FT-516 Avelumab + haNK + IL-15 + vaccine +chemoradiation	I Active I/II Active	/ ORR 67%; PFS(13.7 mths)	locally advanced or metastatic metastatic or unresectable
	PD1/PD-L1 antibody + vaccine	NCT03362060 NCT02432963 NCT03761914	Pembrolizumab+ PVX-410 Pembrolizumab+p53-specifific vaccine Pembrolizumab+WT1-specifific vaccine	I Active I Active I Active	/ / /	metastatic or unresectable advanced or unresectable advanced or metastatic
		NCT03289962 NCT02826434 NCT03199040 NCT03606967	Atezolizumab neoantigen vaccine Durvalumab+PVX-410 Durvalumab+neoantigen DNA vaccine Durvalumab + Nab-paclitaxel + neoantigen vaccine	I Active I Active I Active II Recruiting	/ / / /	locally advanced or metastatic locally advanced metastatic metastatic
	PD1/PD-L1 antibody + OPs	NCT01986426	Pembrolizumab+LTX-315	I Completed	Not Specified	metastatic or unresectable

ORR,overall response rate; OS, overall survival; PFS progression free survival; pCR, pathological complete response;

*PD-L1 cutoff 1%; **PD-L1 cutoff 10%; #BRCA mutant.

AC, doxorubin + cyclophosphamide; EC, epirubicin + cyclophosphamide: FEC, flflurouracil + epirubicin + cyclophosphamide; MEKi, MEK inhibitors; VEGFRi, VEGFR inhibitors; PVX-140, multi-peptide vaccine (XBP1 US184-192: XBP1 SP367-375; CD138260)

FT-156, iPSC-derived NK cells with hnCD16; IL-15, interleukin 15; NK, natural killer cell; POs, Oncolytic peptides.

Chemotherapy can increase tumor cell antigen release; induce the expression of MHC class I molecules, neoantigens, and PD-L1; and promote dendritic cell activation; therefore, it can potentially augment the immune response following or during ICI treatment (53). Based on this rationale, the combination regimens of PD1/PD-L1 inhibitors with chemotherapy have been designed and shown promising results in metastatic, locally advanced and early-stage TNBC (49). The atezolizumab plus nab-paclitaxel won the approval of the FDA for metastatic TNBC, marking the first licensed immunotherapy regimen for breast cancer (54).

PD1/PD-L1 ICI-targeted therapy combination treatment has been investigated in clinical trials. A clinical study using a combination of anti-PD1 antibodies pembrolizumab and the PARPi niraparib reported an ORR of 29% in mTNBC patients with BRCA1/2 mutations (55). Recently, it has been reported that the combination of a deubiquitinase UCHL3 inhibitor, perifosine, and PARP inhibitor Olaparib showed synergistic anti-tumor activity *in vivo* in the TNBC xenograft model (56), suggesting a combination of perifosine, Olaparib, and pembrolizumab should be evaluated in the clinical trial for TNBC in the near future. In addition, several clinical trials have been designed to evaluate the combination of theanti-PD-L1 antibody atezolizumab with PARPi olaparib in mTNBC. Furthermore, a triple-combination treatment of PD-L1 inhibition with PARPi and VEGF inhibitors is currently undergoing. Furthermore, the clinical benefit of combining PARPi, PD1/PD-L1 blockade, and cyclin-dependent kinase (CDK) inhibitors is under evaluation in clinical trials.

Clinical trials using a combination of anti-PD1/PD-L1 ICI with cancer vaccine are ongoing, as well as a combination of anti-PD1/PD-L1 ICI with natural killer cells. In addition, a combination of ICI and ADC may have a synergistic effect and it should be evaluated in clinical trials for TNBC treatment.

Overall, the patients benefitting from ICI treatment are still small compared to other types of cancers with high TMB such as lung carcinoma and myeloma.

Since the TME plays important roles in solid tumor progression, many researchers are trying to identify novel targets in TME to enhance ICI efficacy. Recently, Molgora and colleagues found that Trem2^-/-^ mice are more cancer resistant and more responsive to anti-PD-1 immunotherapy than wild-type mice (57), and treatment with anti-TREM2 mAb combined with anti-PD-1 inhibited tumor growth and promoted tumor regression, with reduced MRC1^+^ and CX3CR1^+^ macrophages in the tumor infiltrate, but the expansion of myeloid subsets expresses immunostimulatory molecules that promote improved T-cell responses. TREM2 might serve as a therapeutic target to modify tumor myeloid infiltrates and augment checkpoint immunotherapy. Transforming growth factor (TGF)-β is an important regulator of immune homeostasis and tolerance, inhibiting the expansion and function of many components of the immune system; a novel approach to enhance ICI treatment on TNBC by targeting the TGF-β pathway in the TME is current undergoing (58).

**Figure 2:**
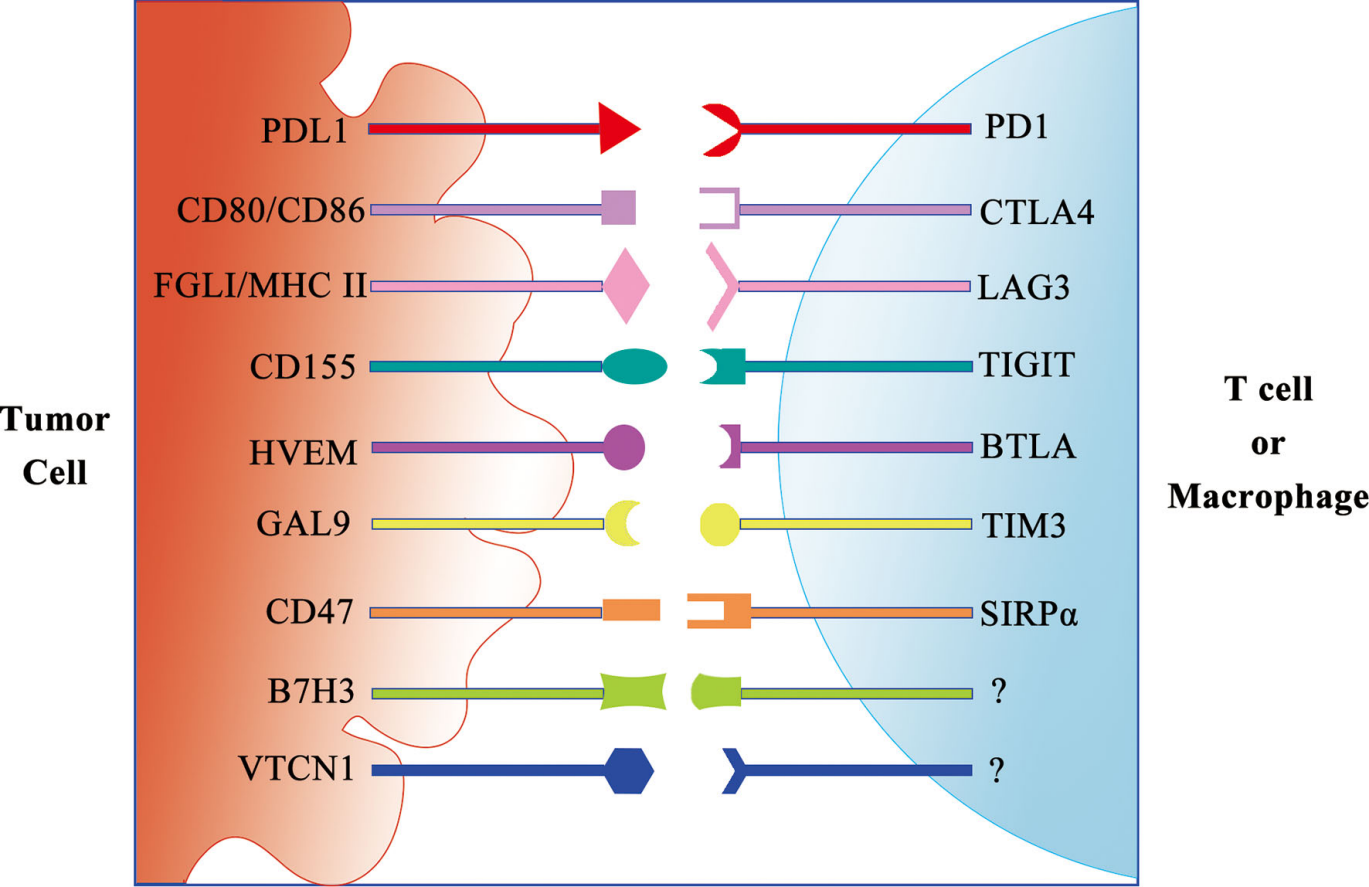
Schematic diagram of the immune checkpoint receptor and its ligand.

In addition to anti-PD-1/PD-L1 and anti-CTLA ICI monotherapy, a few preclinical studies are investigating the benefit of targeting multiple immune checkpoints including PD-1, CTLA-4, Tim3 (CD366, HAVCR2), T-cell immunoreceptor with Ig and ITIM domains (TIGIT), Lag3 (CD223), and B and T lymphocyte attenuator (BTLA) (59) (**Figure 2**).

In addition to ligands to inhibit T cells, the ligands to inhibit other immune cells are discovered. CD47 is a critical self-protective “do-not-eat-me” signal on multiple human cancers against macrophage immunosurveillance (60). The combination of CD47 blockade and macrophage activation by cabazitaxel synergizes to vastly enhance the elimination of TNBC cells. Therefore, targeting macrophages is a promising and effective strategy for TNBC treatment.

In order to benefit from the ICI treatment, the patient’s immune system, particularly the function of T lymphocyte compartment, should be evaluated for patient’s suitability for the administration of ICIs. Not only should the total immune cell numbers be counted, but the proliferation and tumor lytic activities of the T cells should also be measured. If the immune system is severely compromised due to disease progression or previous chemotherapy, an alternative approach such as the adoptive cell transfer and ICI combination should be considered.

### Neoantigen Cancer Vaccine

Neoantigen cancer vaccine can induce an immune response to a tumor-specific antigen (neoantigen) and lead to the generation and expansion of T lymphocytes recognizing tumor antigens and restrict tumor growth. Breast cancer, particularly the TNBC subtype, is immunogenic, and a variety of vaccines have been designed to boost immunity directed against the disease (61). Although several vaccines have advanced to large randomized phase II or phase III clinical trials, none of these trials using cancer vaccine as a single agent met their primary endpoint of either progression-free survival or OS. Therefore, many therapeutic breast cancer vaccines are now being tested in combination with other forms of immune therapy or chemotherapy and radiation.

The success of dendritic cell vaccines targeting HER2-expressing breast cancer proves that vaccination against various onco-drivers can prevent or interrupt the process of breast cancer development (62). In addition, neoantigens also serve as effective targets for interception by the virtue of strong immunogenicity (62).

With the recent approval of two COVID-19 mRNA vaccines (mRNA-1273 and BNT162b2), mRNA vaccines are promising next-generation vaccines that have introduced a new era in vaccinology (63). Recently, mRNA vaccines have become a promising platform for cancer immunotherapy (64). The mRNA cancer vaccine has many advantages compared to other conventional vaccine platforms such as high potency, safe administration, rapid development potentials, and cost-effective manufacturing. With the promising therapeutic outcomes of mRNA cancer vaccines achieved in several clinical trials against multiple aggressive solid tumors, the rapid advancement of mRNA vaccines for cancer immunotherapy is expected in the near future.

The application of the precision or personal medicine is expanding fast due to the much reduced cost of genome sequencing and the advancement of bioinformatics. The neoantigen prediction algorithms are being developed and being validated using different methodologies. With more common neoantigens being identified in tumors, it could lead to more peptide-based or mRNA-based cancer vaccine in combination with other therapies into clinical trials (65).

## Oncolytic Virus-Based Therapy

Oncolytic viruses (OVs) are selectively replication competent in cancer cells and able to amplify themselves after initial administration and potentially spread throughout the tumor, becoming a new class of therapeutic agents that promote anti-tumor responses through a dual mechanism of action that is dependent on selective tumor cell killing and the induction of systemic anti-tumor immunity (66). The talimogene laherparepvec (Imlygic, OncoVEX^GM-CSF^, and T-VEC) is a recombinant herpes simplex virus type 1 (HSV-1) and received FDA approval as the first oncolytic virus for melanoma treatment in 2015.

After T-VEC is approved for melanoma, there are many ongoing clinical trials using T-VEC for other types of solid tumors. In a phase I clinical trial with 30 patients enrolled, T-VEC treatment led to three patients with a stable disease, including one patient with breast cancer, six patients experienced a decrease of tumor size (injected and/or un-injected), including two with breast cancer, and four patients displayed additional inflammation in un-injected lesions. Overall, the results of the clinic studies show that most OVs were found to be safe and well tolerated with few side effects mostly limited to flu-like symptoms or local inflammation at the injection sites. It is still in the early stage and very difficult to draw any reliable conclusions about efficacy, especially with regard to breast cancer patients. It is now only at the beginning of the clinical journey with OVs.

Oncolytic virotherapy can kill tumor cells selectively. In addition, an oncolytic viral infection causes a release of cell debris and antigens to stimulate the immune system (66). A series of processes including viral infection, oncolysis, new antigens, and the activation of cellular danger pathways prevents the tumor from evading the immune system and induces an immune response.

It has been suggested that the subtypes of breast cancer susceptible to ICIs could be sensitized to improve the response to these therapeutic agents such as OV administration. In addition, non-immunogenic tumors can be transformed into immunogenic tumors, thus making them more susceptible to ICIs. OV may fulfill this role and offer a new way of improving ICI treatment. In particular, the activation of an immune response to the tumor cells due to viral infection may play an important part in this approach. For example, it has been reported that a combination of the OV T-VEC with the anti-PD-1 antibody pembrolizumab enhanced the CD8^+^ T-cell count and elevated the PD-L1 protein expression in advanced melanoma, potentially increasing response rates to the ICI (67).

Currently, a few OV candidates other than T-VEC are in the developmental stage. Using a vesicular stomatitis virus (VSV) in combination with an anti-PD-1 checkpoint inhibitor as a therapeutic regime in experimental models of TNBC, Niavarani and colleagues reported that the recruitment of CD8^+^ T cells plays an important role in enhancing the efficacy of immune checkpoint inhibitors (68). PV701, a Newcastle disease virus (NDV)–based OV, is well tolerated, with a partial response in one patient and disease stabilization ≥6 months in four patients with progressive disease in a total of 16 patients enrolled in a phase I clinical trial (69). Pelareorep (Reolysin) is a naturally occurring double-stranded RNA reovirus OV, originated from the serotype 3 Dearing strain. In a phase I clinical trial using pelareorep, there was some evidence of local target tumor response activity in 7 of 19 patients with one breast cancer patient exhibiting a stable disease after six or more weeks (70). HF10, an OV based on HSV-1 strain, can induce cell death occurring in 30%–100% of malignant cells in patients injected with HF10, whereas no cell death was observed in the saline-injected nodules in a phase I clinical trial (71). JX-929 (vvDD) is a genetically engineered Western Reserve strain vaccinia virus with two gene deletions, and it appears safe for use in patients and shows selective replication in injected and un-injected tumors in a phase I clinical trial (72).

Adenovirus (Ad) serotypes 2 and 5 are currently been evaluated as oncolytic adenoviruses with a few ongoing clinical trials for the treatment of breast cancer (73). In a phase I clinical trial with 12 patients available for follow-up, treatment with ICOVIR-7, an adenovirus with a genetical deletion allowing the regulation of a gene by a tumor-specific promoter E2F-1, leads to two stable diseases, two minor responses, and one partial response, including one of the patients with breast cancer exhibiting a decrease or stabilization of tumor markers (74). In a phase I clinical trial with 12 patients enrolled, Telomelysin (OBP-301), a genetically modified serotype 5 adenovirus-based OV, led to one partial response, and 7 stable diseases occurred at a follow-up 56 days after treatment (75). In a phase I trial investigating the combination of adenovirus ONYX-015/dl1520 (lontucirev) together with the synthetic dimer of the human TNF-α receptor etanercept in patients with solid tumors including two patients with breast cancer, the combination therapy led to 4 of 9 patients showing a stable disease (76). In a phase I clinic trial, a combination of Ad5/3-D24-GMCSF (CGTG-102) OV and low-dose cyclophosphamide was administered to 16 patients with advanced breast cancer and found to be well tolerated with the evidence of tumor shrinkage in 3 of 14 imaged patients (77). In a phase I clinical trial with 12 patients enrolled using an OV H103, a recombinant oncolytic serotype 2 adenovirus overexpressing heat shock protein 70 (Hsp70), three patients showed a partial or complete response in the original tumor and another three patients also displayed a response in metastases not injected with the OV (78).

In addition to the combination therapies of OVs with ICI, the combination of OVs with CAR-T are in the developmental stage to treat solid tumors such as TNBC.

## Adoptive Cell Transfer–Based Therapy

### Tumor-Infiltrating Lymphocyte

**Figure 3:**
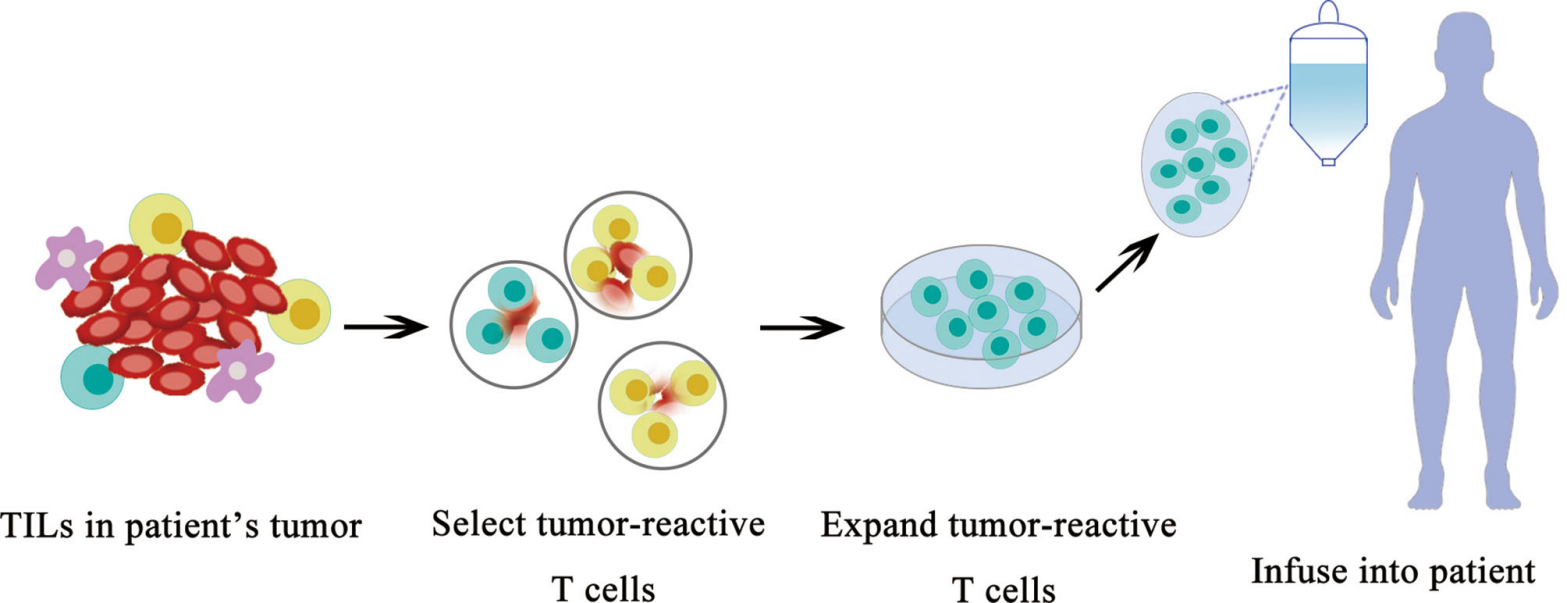
Schematic diagram of TIL therapy.

TILs have the ability to recognize and kill tumor cells. However, the number of TILs in a tumor is limited and their functions are usually suppressed by the tumor or tumor microenvironment; therefore, they are unable to control tumor growth. Nevertheless, TILs can be expanded to a large quantity using *in vitro* culture, and they can eliminate tumors when they are re-introduced into patients.TIL adoptive cell transfer (ACT) was originally designed to treat melanoma with high mutational burden (79). TILs are usually collected from resected tumor material; then, neoantigen-specific TILs are enriched and expanded *ex vivo* and delivered back to the patient as therapeutic agents. ACT with TILs has been shown to cause objective tumor regression in several types of cancers including melanoma, cervical squamous cell carcinoma, and cholangiocarcinoma (80, 81). The roles of TILs in TNBC are recently investigated in basic and clinical research (**Figure 3**).

TIL was originally examined in TNBC for diagnostic purposes. Later, TIL in TNBC represents the first biological prognostic biomarker for early-stage TNBCs (82), and emerging data suggest that the TIL quantity can help clinicians identify patients with breast cancer who benefit most from PD-1/PD-L1 inhibition.

In addition to help clinicians make a treatment plan, the therapeutic potentials to use TIL to treat breast cancer were recently revealed. Zacharakis and colleagues reported that one patient with metastatic breast cancer who was treated with TILs reactive against the mutant versions of four proteins (SLC3A2, KIAA0368, CADPS2, and CTSB) in conjunction with IL-2 and the checkpoint blockade mediated the complete durable regression of metastatic breast cancer for >22 months, revealing a novel immunotherapy approach for the treatment of breast cancers (83). After the initial case report of treatment using TIL, Zacharakis and colleagues reported a preliminary result of a pilot trial of mutation-reactive TILs in patients with metastatic breast cancer (84). They were able to isolate and grow TILs in culture from the resected lesions of all 42 patients. Twenty-eight of 42 (67%) patients contained TILs recognizing at least one immunogenic somatic mutation, and 13 patients demonstrated robust reactivity for ACT. Six patients were lately enrolled on a protocol of the ACT of enriched neoantigen-specific TILs, in combination with the anti-PD-1 antibody pembrolizumab (≤4 doses). Objective tumor regression was observed in three patients, including one complete response (now ongoing over 5.5 years) and two partial responses (6 and 10 months). They concluded that breast cancer patients generated a natural immune response to their cancer mutations and the adoptive transfer of TIL is a highly personalized experimental option for those patients. This clinical trial demonstrates a great therapeutical potential to use neoantigen-specific TIL to treat breast cancer in the near future (**Figure 3**).

In addition to the adoptive transfer of TILs, the ACT of dendritic cells, natural killer cells, and engineered immune components such as CAR constructs and engineered T-cell receptors (TCRs) are continuously improved with overwhelmingly technical advances in traditional challenges such as toxicity, adoptive cell persistence, and intra-tumoral trafficking (85).

### Chimeric Antigen Receptor T Cells

Similar to a T-cell engager, CAR-T cells can recognize a tumor antigen by its expression of CARs and release cytotoxicity against tumor cells.

**Figure 4:**
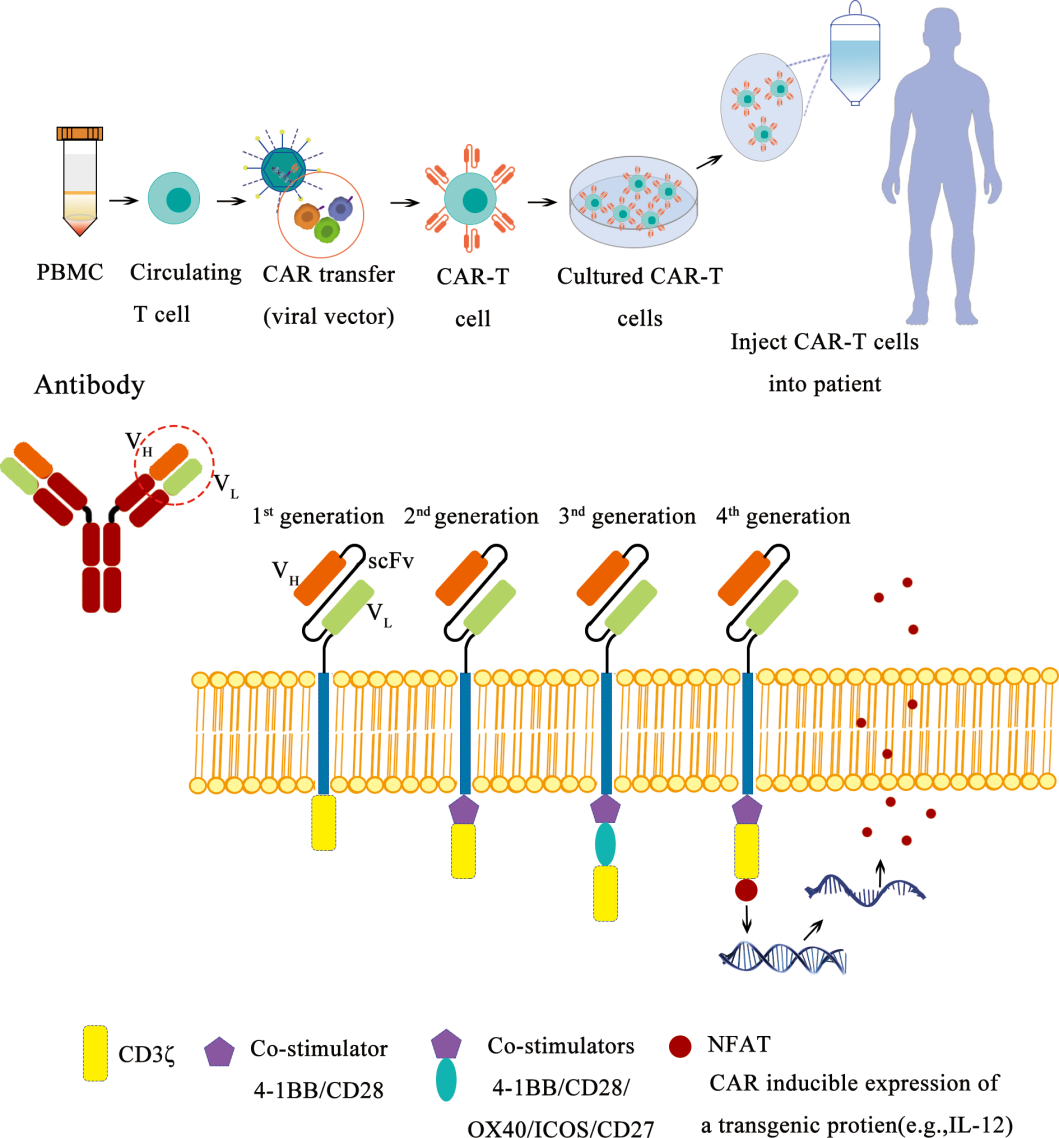
Schematic diagram of CAR-T therapy.

Although the therapeutical potential of CAR-T therapy has been demonstrated in hematological malignancy with the FDA approval of Kymriah and Yescarta, two second-generation CAR-T cell products targeting the B-cell antigen CD19 in 2017, the application of CAR-T therapy on TNBC is still waiting to be evaluated in well-designed clinical trials (**Figure 4**). There are a few factors to be considered to improve CAR-T efficacy for solid tumors such as breast cancer, the target molecule (tumor antigen), cell-intrinsic and -extrinsic factors for T-cell persistence and homing, the tumor microenvironment, toxicities, and management as well as universal CAR-T and combinatorial CAR-T therapy approaches (86–90). With more factors identified to improve the CAR-T therapy, more clinical trials will start to evaluate these options to establish a more potent CAR-T treatment on TNBC. Currently, there are a few promising targets for CAR-T cell therapy in TNBC, including EGFR, EpCAM, GD2, ROR1, AXL, MUC1, CSPG4, FRα, ICAM-1, integrin αvβ3 or αvβ6, NKG2D, SSEA-4, TEM8, mesothelin, c-Met, TROP2, CD44v6, and Fc-gamma receptor (FcγR).

EGFR is a receptor tyrosine kinase that promotes the growth, survival, and invasion of cancer cells. It is estimated that 45%–70% of patients with TNBC overexpress EGFR, and anti-EGFR CAR-T cells are currently being evaluated for TNBC treatment as monotherapy or combination therapy (91, 92). In addition, the expression of EGFR variant III (EGFRvIII) is tumor restricted, and it has been reported that EGFRvIII-specific CAR-T cells have reduced immune exhaustion and enhanced anti-glioma therapeutic function with a lower risk of on-target/off-tumor toxicity mediated by the CAR recognizing antigens on normal tissue (93).

Epithelial cell adhesion molecule (EpCAM) is a cell surface molecule involved in cell-to-cell adhesion, and it is overexpressed 100- to 1,000-fold in primary and metastatic breast cancer, and a phase I clinical trial is currently investigating third-generation EpCAM-CAR-T cells for the treatment of breast cancer (94).

Disialoganglioside (GD2) is a glycosphingolipid that facilitates the tethering of tumor cells to extracellular matrix proteins and highly expressed in stem-like CD44^high^ CD24^low^ human breast cancer cells. Third-generation CAR-T cells have been engineered with an scFv derived from anti-GD2 dinutuximab to target TNBC cells, showing anti-cancer activity and increased persistence in an orthotopic xenograft mouse model of human TNBC (95).

Receptor tyrosine kinase-like orphan receptor 1 (ROR1) has demonstrated to be highly expressed in a subset of TNBC. A ROR1-CAR T cells have the anti-tumor function on TNBC cell line MDA-MB-231. In addition, the anti-tumor function of the ROR1-CAR T cells can be enhanced with SD-208, a highly selective, competitive, and orally bioavailable TGF-β-receptor I kinase inhibitor in a microphysiologic 3D TNBC model (96).

AXL is a receptor tyrosine kinase involved in tumor progression, and the overexpression of AXL on tumor cells is correlated with poor prognosis in several cancers including TNBC (97). AXL has emerged as a therapeutic drug target for TNBC treatment, and AXL-CAR-T cell therapy led to significant *in vitro* cytotoxicity and cytokine secretion as well as a reduction in tumor growth in an TNBC xenograft mouse model (98). Furthermore, AXL-CAR-T cells may be able to overcome the immunosuppressive TME by inhibiting the release of suppressive chemokines and cytokines from tumor-associated microphage (TAM) and by causing myeloid-derived suppressor cell (MDSC) depletion from the TME, fundamentally altering the TME to a proinflammatory state (98–100). More recently, *in vitro* findings supported an anti-tumor activity and prolonged survival for IL-7-expressing AXL-CAR-T cells in a TNBC subcutaneous xenograft model (101), indicating the importance of AXL as a novel CAR-T cell therapy target in TNBC and its potential to modulate the TME to a proinflammatory state for effective anti-tumor immune responses.

Mucin1 glycoprotein (MUC1) is a transmembrane protein protecting epithelial cells from infection by serving as a protective mucosal barrier. An aberrantly glycosylated tumor form of MUC1 (tMUC1) has been found overexpressed in greater than 95% of all TNBC, while no significant tMUC1 expression is detected on normal breast tissues, suggesting a tumor-specific antigen target in TNBC treatment. Recently, second-generation tMUC1-CAR-T cells demonstrated potent tumor cytolytic activity and cytokine production *in vitro* and significant tumor inhibition in a TNBC xenograft mouse model (102).

The chondroitin sulfate proteoglycan 4 (CSPG4) has been identified as a target for many cancers including melanoma, leukemia, glioblastoma, and TNBC. It has been reported that a major advantage of CSPG4-CAR-T cell therapy is its ability to target both primary TNBC cells and cancer-associated fibroblasts (CAFs) because CSPG4 is highly expressed on stromal cells in the TNBC TME (103). Since CSPG4-CAR-T cells can target various molecules simultaneously, including TNBC cells, stromal cells, and blood vessels, a primary safety issue involving its potential on-target, off-tumor toxicity, should be carefully monitored, especially in the form of severe bleeding.

Folate receptor alpha (FRα) is a glycosylphosphatidylinositol (GPI)-linked membrane protein that binds to and mediates the intracellular transport of folate, and FRα is overexpressed in cancers of epithelial origin including lung, colorectal, ovarian, and breast tumors, correlated to a poor prognosis. Therefore, FRα has been identified as an attractive anticancer therapeutic target for TNBC (104). Coherently, FRα-CAR-T cells have shown significant anti-cancer activity in TNBC cell lines and in an MDA-MB-231 xenograft mouse model, which is correlated with FRα expression levels on tumor cells (105). A phase I clinical trial of FRα-CAR-T cells is undergoing.

Intercellular adhesion molecule 1 (ICAM-1) is a surface adhesion molecule highly expressed on TNBC cells; affinity-variant CD28/4-1BB co-stimulated ICAM-1-CARs have recently demonstrated that lower affinity has superior anti-tumor efficacy, with acceptable safety, compared to their higher-affinity counterpart (106, 107). Overall, ICAM-1-CAR-T cells showed significant cytotoxicity against TNBC cells, providing a rationale for early-phase clinical development.

Second-generation anti-integrin αvβ3 CAR-T cells with an EGFRt safety switch have been generated and have demonstrated potent anti-TNBC functions *in vitro*, and the CAR-T cells can be rapidly depleted through endogenous ADCC mechanisms to prevent unwanted toxicity upon the administration of the anti-EGFR mAb cetuximab (108). Moreover, integrin αvβ3-CAR-T cells have the potential to shift the TME to a proinflammatory state because the adhesion molecule is highly expressed in the stromal compartment, suggesting integrin αvβ3 as an important TNBC target for CAR-T cells (108).

The αvβ6 integrin is strongly upregulated in multiple solid tumors and is associated with poorer prognosis in several cancers, exerting pro-tumorigenic activities including the promotion of tumor growth, migration, and invasion. Because the physiologic expression of αvβ6 is largely restricted to wound healing, this epithelial-specific integrin becomes a highly attractive candidate for targeting using immunotherapeutic strategies such as CAR-T immunotherapy (109).

Natural killer group 2D (NKG2D)-CAR-T approaches have demonstrated significant anti-tumor activity in TNBC both *in vitro* and *in vivo* and are currently being investigated in early-phase clinical trials, beginning with hematologic malignancies and metastatic colorectal cancer (110).

The expression of stage-specific embryonic antigen-4 (SSEA-4) is limited in normal tissues and upregulated in approximately 30% of TNBC tumor cells. Second-generation SSEA-4-CAR-T cells have been demonstrated to inhibit TNBC cells *in vitro* and in an MDA-MB-231 xenograft mouse model (111). Since hematopoietic multipotent progenitor cells in the bone marrow and epithelial pluripotent cells in the lungs are cotargeted by the SSEA-4-CAR-T cells due to low-level antigen expression, the safety mechanisms should be considered to avoid on-tumor/off-target toxicities and potential life-threatening side effects.

Tumor endothelial marker 8 (TEM8) is a cell surface protein that is preferentially expressed in areas of aberrant neoangiogenesis within tumors. Second-generation (CD28/CD3ζ) and third-generation (CD28/4-1BB/CD3ζ) TEM8-CARs have been engineered to co-target TNBC cells expressing TEM8 as well as tumor-associated vessels, demonstrating the ability to induce TNBC cell regression, as well as to reduce tumor neoangiogenesis in the xenograft mouse model (112, 113).

In addition, mesothelin-CAR-T cells, c-MET-CAR-T cells, TROP2-CAR-T cells, and CD44v6-CAR-T cells have been engineered for the treatment of TNBC (88).

A universal CAR-T cell that expresses an Fc-gamma receptor (FcγR)-CAR such as CD16A158F and CD32A131R CARs has been generated, using multiple therapeutic antibodies to redirect T cells to antigen-expressing tumor cells including EGFR-overexpressing TNBC (114, 115). Since therapeutic antibodies that target antigens on solid tumors are available, the use of FcγR-CAR-T cells in combination with these antibodies is a viable option to eliminate solid tumors including TNBC.

Novel CAR-T cells that incorporate ICIs such as anti-PD-1 and anti-CTLA-4 into EGFR-CAR-T cells are also being studied in the clinic. Since CAR-T cells will secrete these ICI antibodies, this treatment modality will serve as combination therapy and may modulate the immunosuppressive TME (116).

A novel strategy to avoid possible off-tumor toxicity is to generate a synthetic Notch (synNotch) receptor (117, 118). The engineering of ROR1-CAR-T cells with synNotch receptors that are specific for EpCAM or B7-H3, expressed on ROR1^+^ tumor cells but not on ROR1^+^ stromal cells, can induce ROR1 expression selectively within the tumor, thus sparing normal tissues (119).

Recently, Narayan and colleagues reported results from an in-human phase I trial of castration-resistant, prostate cancer-directed CAR-T cells armored with a dominant-negative TGF-β receptor (120). CAR-T cell kinetics revealed expansion in blood and tumor trafficking, proving that a clinical application of TGF-β-resistant CAR-T cells is feasible and generally safe, suggesting a novel strategy to improve therapy outcomes by a superior multipronged approach against the TME.

The identification of TNBC-specific antigens such as ROR1 and AXL could lead to an efficient CAR-T therapy approach to treat TNBC, similar to CD19 CAR-T for pre-B ALL treatment.

### Chimeric Antigen Receptor Natural Killer Cell

Similar to CAR-T, CAR-NK cells can recognize a tumor antigen by its expression of CARs and release cytotoxicity against tumor cells.

Bendelac and colleagues reported the identification and characterization of the natural killer T cell, a lymphocyte that is able to bind and kill certain tumor and virus-infected cells in 1994. Later, the ACT with isolated mature or *in vitro* differentiated NK cells without engineering from different sources, including peripheral blood mononuclear cells (PBMCs), a newborn body’s umbilical cord blood or placenta, or induced pluripotent stem cells (iPSs) have demonstrated limited anti-tumor ability *in vivo*.

Encouraged by the successes of CAR-T in cancer treatment, the chimeric antigen receptor natural killer cells (CAR-NKs) are developed as another engineered immune cells for adopting cell transfer to treat cancers. Compared to CAR-T, CAR-NK can have a few advantages. It does not require MHC-matching; therefore, the NK can have a few additional sources other than patients themselves. NK from peripheral blood, cold blood, or placenta or that are iPS derived are all considered to be the resource of NK cells. NK could have a less adverse effect due to moderate cytokine release compared to CAR-T (121–123).

It has been recently demonstrated that HER2 CAR-expression in NK cells from healthy donors and patients with breast cancer potently enhances their anti-tumor functions against various HER2-expressing cancer cells, regardless of MHC class I expression (124). Moreover, HER2 CAR-NK cells exert higher cytotoxicity than donor-matched HER2 CAR-T cells against tumor targets. Importantly, unlike CAR-T cells, HER2 CAR-NK cells do not elicit enhanced cytotoxicity or inflammatory cytokine production against non-malignant human lung epithelial cells with basal HER2 expression. Further, HER2 CAR-NK cells maintain a high cytotoxic function in the presence of immunosuppressive factors enriched in solid tumors. These results show that CAR-NK cells may be a highly potent and safe source of immunotherapy in the context of solid tumors.

Currently, CAR-NK has been vigorously tested in breast cancer treatment targeting different breast cancer antigens including HER2 and EGFR, and EGFR-CAR-NK cells could be potentially used to treat patients with TNBC exhibiting enhanced EGFR expression (125). In addition, it has been reported that PD-L1-CAR-NK cells with PRDX1 overexpression display a potent antitumor activity against breast cancer cells under oxidative stress (126). HLA-G CAR-NK cells present an effective cytolysis of breast, brain, pancreatic, and ovarian cancer cells *in vitro*, as well as reduced xenograft tumor growth with extended median survival in orthotopic mouse models (127). A preclinical report demonstrated that the tissue factor (TF)-CAR-NK cells alone could kill TNBC cells and their efficacy were enhanced with L-ICON ADCC *in vitro* (128). Moreover, TF-CAR-NK cells were effective *in vivo* for the treatment of TNBC in cell line- and patient tumor-derived xenograft mouse models (128). Recently, it has been demonstrated that the regional administration of EGFR-CAR NK-92 cells combined with oHSV-1 OV therapy is a potentially promising strategy to treat TNBC (129).

The identification of TNBC-specific antigens such as ROR1 and AXL could also lead to an efficient CAR-NK therapy approach to treat TNBC, similar to CD19 CAR-T for pre-B ALL treatment.

### Chimeric Antigen Receptor Macrophage

Similar to CAR-T or CAR-NK, CAR-M cells can recognize a tumor antigen by its expression of CARs and release cytotoxicity against tumor cells.

Macrophages are innate immune cells that are intrinsically equipped with broad therapeutic effector functions, including active trafficking to tumor sites, direct tumor phagocytosis, the activation of the tumor microenvironment, and professional antigen presentation. Among cell types used in immunotherapies, however, macrophages have recently emerged as prominent candidates for the treatment of solid tumors. Similar to CAR-NK, engineered CAR-M is proposed to treat solid tumors. In addition to killing tumor cells, CAR-M is predicted to serve as antigen-presenting cells to stimulate the immunity and activation of the tumor microenvironment. CAR-M has demonstrated antigen-specific phagocytosis and tumor clearance *in vitro* (130–133). In two solid tumor xenograft mouse models, a single infusion of human CAR-Ms decreased tumor burden and prolonged OS. In humanized mouse models, CAR-Ms were further shown to induce a pro-inflammatory TME and boost anti-tumor T-cell activity. CAR-M therapies are able to clear tumor cells *in vitro* and in preclinical *in vivo* models. It has been demonstrated that human CAR-Ms exhibit antigen-specific phagocytosis, cytokine/chemokine secretion, and the killing of antigen-expressing targets *in vitro* (134).

The CAR-M has been investigated to treat breast cancer. Recently, Pierini and colleagues reported that they have established an immunocompetent, syngeneic CAR-M model and demonstrated that murine CAR-M increased intratumoral T-cell infiltration, NK-cell infiltration, dendritic cell infiltration/activation, and TIL activation (135). They found that CAR-M locally administered in HER2^+^ tumors simultaneously controlled the growth of contralateral HER2-negative tumors and prevented antigen-negative relapse upon an HER2-negative tumor rechallenge, indicating epitope spreading and the induction of long-term immune memory. Notably, this work also demonstrated for the first time that CAR-Ms synergize with PD1 blockade in PD1-monotherapy-resistant solid tumor models (135). In two immunodeficient NSGS xenograft models, a single dose of anti-HER2 CAR-M reduced tumor burden and prolonged OS against HER2^+^ SKOV3 tumors.

The efficacy of CAR-M has to be evaluated in the clinical trials. Meanwhile, the manufacture of CAR-M has to be improved.

### T-Cell Receptor T Cells

Similar to CAR-T, TCR-T cells can recognize the tumor antigen presented by the MHC complex by its expression of TCRs and release cytotoxicity against tumor cells.

**Figure 5:**
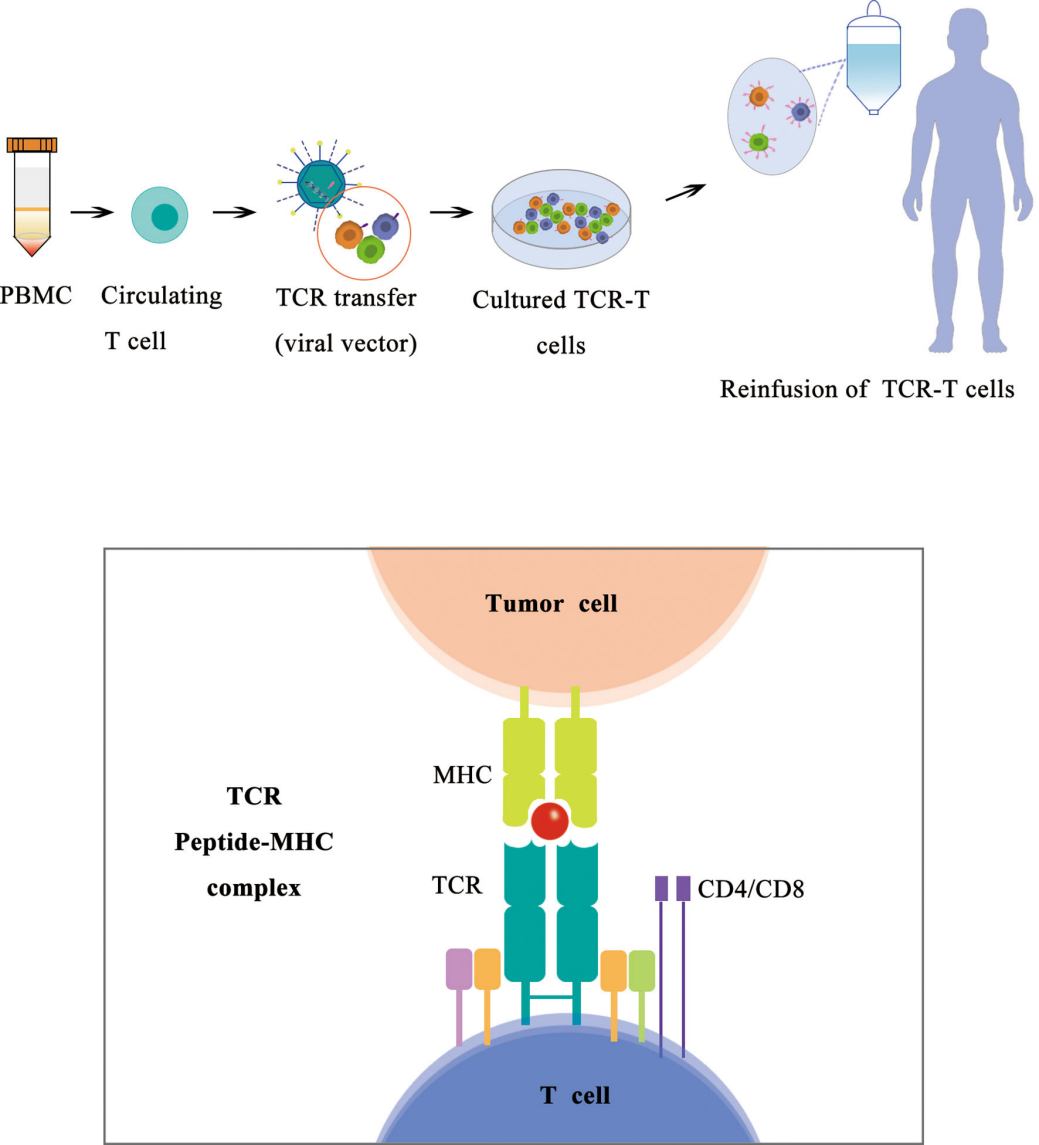
Schematic diagram of TCR-T therapy.

With most recent approval of Kimmtrak (tebentafusp) in 2022, TCR-T therapy becomes a frontier in cancer treatment using ACT. Building upon advances in TCR isolation and gene-engineering technologies, TCRs recognizing a wide range of specific peptide/HLA combinations can now be expressed in a patient’s T cells to generate TCR-transgenic T (TCR-T) cells, redirecting those cells to recognize tumor-associated antigens and kill tumor cells (136–139). Adoptively transferred TCR-T cells are not restricted by the cell surface expression of their targets and are therefore poised to become a main pillar of cellular cancer immunotherapies. Because TCR gene transfer confers a novel specificity to the treated patient’s T cells, TCR-T cells do not rely on a patient’s preexisting endogenous T-cell repertoire and can help overcome resistance to ICIs. TCR-T cells are also not restrained by the limited availability of cancer-specific cell surface proteins that are required for successful targeting by chimeric antigen receptor (CAR)–engineered T cells (**Figure 5**).

There are three major subtypes of TCR-Ts according to their targets. A subset of TCR-T targets viral antigens, a subtype of TCR-T targets tumor-specific mutated proteins (neoantigen), and a subtype of TCR-T targets tumor-associated antigens including cancer/testis (CT) antigens, overexpressed antigens, and differentiation antigens. Among all the target antigens, most clinical trials of TCR-T cell therapy have targeted CT antigens and viral antigens, with New York esophageal squamous cell carcinoma-1 (NY-ESO-1) being the most frequently targeted to date (139).

Similar to other ACT approaches, the TCR-T approach encountered many challenges in preclinical optimization and clinical translation including the selection of target antigens, tumor antigen heterogeneity and tumor immune escape, the off-target and safety problems during TCR gene transfer, T-cell unresponsiveness and exhaustion, and toxicity caused by cytokine storms.

The optimization of TCR-T therapy is a complex interdisciplinary issue that integrates immune-oncology, tumor biology, and genetic engineering, including the systemic selection of the TCR-T target antigen, the counteracting influence of tumor antigen heterogeneity, the development of a safer TCR gene transfer method, and the development of new gene engineering approaches to enhance TCR-T-cell function and optimize anti-tumor immune responses.

In terms of target selection, the immune selection pressure may lead to the downregulation of target antigens, reducing the efficacy of TCR-T-cell therapy, and the loss of targeted tumor antigens may result in tumor recurrence even after the infusion of adoptive functional cells. The target antigen downregulation may be overcome by different strategies including targeting proteins with core functions in tumor survival, infusing multiple T-cell clones with different tumor-specific TCRs or infusing T cells targeting two or more tumor antigens (139). Novel technologies based on high-throughput sequencing along with progress in bioinformatics have been advanced to overcome the challenges of antigen selection in TCT-T therapy. For example, the company Kite Pharma developed a high-throughput sequencing platform for the immune repertoire (HTS-IR) and computational biology methods including TraCeR and single-cell TCRseq at the population and single-cell levels to reconstruct TCR and identify immunogenic neoantigens. Another example, a flow cytometry-based method, has been developed to select tumor antigen–specific T cells from patients, with TCR genes that recognize these antigens obtained by single-cell technology and introduced into patients’ peripheral T cells for treatment. Those new tools have been utilized for analyzing the diversity and dynamics of T cells.

In terms of tumor antigen heterogeneity, the expression level of tumor antigens varies in different cells within tumors, allowing some tumor cells with lower antigen expression to escape from specific antigen-targeted therapy and leading to therapeutic resistance in adoptive T-cell therapy. This issue could be overcome by the construction of TCR-T cells with common new antigens, new antigens covering most tumor subclones, or new antigens from driver mutations. In addition, increasing the structural affinity of TCR may enhance their anti-tumor activity, including selective modifications to the CDR3 region of TCR α and β chains for antigen recognition and binding, the codon optimization of TCR to increase protein expression, reducing the glycosylation of TCR, modifying three transmembrane residues of the TCR α chain to hydrophobic amino acids to enhance the anti-tumor functional affinity of T cells and to increase the stability and level of expression of TCR, and the design of the gene expression box using P2A or IRES elements linking α and β chains to increase TCR expression levels and reduce the risk of inducing autoimmune pathological changes (140).

In terms of a safer TCR gene transfer method, TCR-T cells can form a complex of four different TCRs, with two chains derived from exogenous α/β TCRs and the other two from endogenous α/β TCRs. These heterodimeric TCRs can form a receptor with new specificity or a nonfunctional complex and may cause an unfavorable response. Many strategies have been developed to overcome this problem, including a CRISPR-Cas9 gene editing approach to remove endogenous TCR or an siRNA-based approach to silence endogenous TCR gene expression.

In terms of the enhancement of TCR-T-cell function and the optimization of anti-tumor immune responses, solid tumors are characterized by a complex immunosuppressive microenvironment including tumor cells, fibroblasts, immune cells, signaling molecules, and extracellular matrices, inhibiting TCR-T-cell function, significantly affecting tumor diagnosis, patient survival, and treatment sensitivity. To enhance TCR-T function and optimize its anti-tumor immune response, tumor-associated T-cell dysfunction and exhaustion have to be adjusted, including the physical removal of dysfunctional cells from the circulation to ensure the homeostatic proliferation of effector and memory T cells by autologous stem cell transplantation (ASCT); the reprogramming and redifferentiation of induced pluripotent stem cells originally derived from T cells (T-iPSCs) or their dedifferentiation within their own lineages (141); or the recovery method to restore and maintain the thymus environment by bioengineering thymic organoid substances, growth-promoting factors, and cytokines (particularly IL-21) and to further reverse thymus degeneration.

Although TCR-T therapy encounters many challenges in the treatment of solid tumors, a recent report by Yarmarkovich and colleagues to treat brain tumor may lead to success in the treatment of solid tumors including TNBC in the future (142). Since the majority of oncogenic drivers are intracellular proteins, the discovery of the cognate immunotherapeutics targeting mutated peptides (neoantigens) presented by individual human leukocyte antigen (HLA) allotypes is thus constrained. Therefore, most cancers have a modest tumor mutational burden that is insufficient to generate responses using neoantigen-based therapies. Neuroblastoma is a pediatric cancer with very low TMB but driven by epigenetically deregulated transcriptional networks. These authors found that the neuroblastoma immunopeptidome is enriched with peptides derived from proteins that are essential for tumorigenesis and focused on targeting the unmutated peptide QYNPIRTTF, discovered on HLA-A*24:02, derived from the neuroblastoma master transcriptional regulator PHOX2B. To target QYNPIRTTF, they developed peptide-centric CARs using a counter-panning strategy, further demonstrating that peptide-centric CARs could recognize peptides on additional HLA allotypes when presented in a similar manner. Informed by computational modeling, they reported that PHOX2B peptide–centric CARs also recognize QYNPIRTTF presented by HLA-A*23:01 and the highly divergent HLA-B*14:02. Finally, they demonstrated the potent and specific killing of neuroblastoma cells expressing these HLAs *in vitro* and complete tumor regression in the mouse model. These data suggest that peptide-centric CARs have the potential to vastly expand the pool of immunotherapeutic targets to include non-immunogenic intracellular oncoproteins and widen the population of patients who would benefit from such therapy by breaking conventional HLA restrictions, a novel TCR-T therapy platform.

## Challenges and Opportunities in TNBC Immunotherapy

Cancer immunotherapy has become the standard treatment for many cancers, including melanomas and lung cancers, and is now the fifth pillar of cancer therapy. However, only a small fraction of TNBC patients benefit from immunotherapy.

There are at least three challenges in TNBC treatments using the immunotherapy approach (143–145). The specific targets of TNBC are limited. The activity of immune cells is affected by the TME. The intrinsic and extrinsic regulators of immune cells are mostly unknown. To overcome these challenges, both basic research and translational research are required. New knowledge on tumor-immune interaction in TME is revealed based on emerging novel technologies such as high-throughput single-cell sequencing and proteomics, leading to the opportunity of cancer therapy eventually (146).

Using the targeted capture and long-read sequencing of TCR and B-cell-receptor (BCR) mRNA transcripts with short-read transcriptome profiling of barcoded single-cell libraries generated by droplet-based partitioning, and a bioinformatics method named Repertoire and Gene Expression by Sequencing (RAGE-Seq), which can generate accurate full-length antigen receptor sequences at nucleotide resolution, infer B-cell clonal evolution and identify alternatively spliced BCR transcripts (147), Singh and colleagues are able to analyze the clonal and transcriptional landscapes of lymphocytes, revealing lymphocyte dynamics.

Using a different approach, Azizi and colleagues proposed a model of continuous activation in T cells but not the macrophage polarization model in cancer (148) by the analysis of paired single-cell RNA and TCR sequencing data from 27,000 additional T cells, drawing a single-cell atlas of diverse immune phenotypes of breast cancer samples and found that the immune phenotype was associated with the tissue of residence.

Savas and colleagues recently reported that a tissue-resident memory (TRM) T-cell phenotype in breast cancer using the single-cell sequencing approach (149). They found that a CD8^+^CD103^+^ cell population has a highly distinct gene expression including several hallmarks of TRM differentiation with a high expression of both immune checkpoint molecules (such as PD-1 and CTLA4) and cytotoxic effector proteins (such as GZMB and PRF1) when compared with the other T-cell clusters. Further, the gene signatures of the CD8^+^CD103^+^ TRM cluster were found to be significantly correlated with favorable patient survival in early-stage TNBC. This study suggests that scRNA-seq can lead to the discovery of minor subgroups of TILs that were related to immune suppression or immune surveillance, and the biomarkers of these distinct immune cells may serve as prognostic factors or therapeutic targets for breast cancer (149).

In another study, Lu and colleagues observed a phenotype switch of B cells during neoadjuvant chemotherapy that could enhance tumor-specific T-cell responses (150). They identified a distinct B-cell subset with high levels of inducible T-cell co-stimulator ligand (ICOSL) significantly increased after neoadjuvant chemotherapy. In addition, the high expression of CR2 and the low expression of IL-10 were also found in this special B-cell subset. They found that ICOSL^+^ B-cell abundance was an independent positive prognostic factor and related to improved therapeutic efficacy. They also found the CD55 expression on tumor cells as the key factor in determining the ICOSL^+^ B subset switch and conflicting roles of tumor-infiltrating B cells during chemotherapy. They proposed that this chemotherapy-associated subset of B cells could promote tumor-specific T-cell proliferation and reduce regulatory T cells (Tregs). In summary, this study uncovered a new role of complement in B-cell-dependent anti-tumor immunity and indicated that CD55 induced chemo-resistance by impeding the induction of ICOSL^+^ B cells and could thus be a potential therapeutic target to enhance the efficacy of immunogenic chemotherapy.

All these findings based on scRNA-seq analysis offered a more nuanced view into the association between immune phenotypes and the tissues of residence and suggested that the immunological landscape based on the blood or normal samples may not reflect the functional and phenotypic diversity in the TME (146).

One of major challenges in ACT therapy is to maintain the proliferative and functional activity of immune cells during expansion *ex vivo* and after transfer *in vivo*. The identification of novel intrinsic factors to maintain the stemness phenotype can lead to the improvement of CAT-T function. Recently, a few genome-wide screens show potentials to identify intrinsic factors to enhance CAR-T activities.

Using optimized lentiviral methods to enable efficient and scalable delivery of the CRISPRa machinery into primary human T cells, Schmidt and colleagues performed genome-wide pooled CRISPRa screens to identify the critical regulators of cytokine production (151). They found that IFN-γ production is strongly regulated by the nuclear factor κB (NF-κB) signaling pathway and the overexpression of receptors such as 4-1BB, CD27, CD40, and OX40 can promote IFN-γ production and enhance T-cell function. The identification of critical regulators in T-cell function can provide novel strategies for improving next-generation T-cell therapies.

Using a barcoded human open reading frame (ORF) overexpression system, Leget and colleagues examined the functions of T cells with the overexpression of a pool of approximately 12,000 ORFs, and identified the lymphotoxin-β receptor (LTBR) as a top hit that is typically expressed in myeloid cells but absent in lymphocytes (152). They found that LTBR overexpression in T cells can increase T-cell effector functions and resistance to exhaustion by the activation of the canonical NF-κB pathway. LTBR can improve CAR-T function and may offer opportunities to engineer next-generation immunotherapies.

The complexity feature of TME suggests that the combinatory approach using cancer genomics and adoptive cell transfer may be a way to be explored in the future. Immunotherapy can also be applied in combination with traditional surgery procedures. Chemotherapy is usually followed after surgery to eliminate potential metastasis. However, Neo-antigen-based vaccine could provide an alternative. Considering the maturation of the design and the manufacture of mRNA-based vaccine and personal genomic sequencing, the mRNA-based cancer vaccine against Neo-antigen can be applied after tumor surgery removal and tumor genomic sequencing to replace the chemotherapy with less adverse effects. In addition, combined agents that stimulate migration, inflammation, and tumor barrier dissolution with CAR-based therapy will provide critical avenues for advancing engineered T- and NK-cell therapy against solid tumors (153).

Another progress in gene editing could provide a more efficient CAR-T manufacturing procedure. The current approach of CRISPR, TALENT or zinc finger nuclease has at least two concerns: the delivery efficiency and off-target possibility. While the viral-vector based delivery is more efficient, its off-target possibility is increased. On the other hand, transient protein delivery reduces the off-target possibility but has low efficiency and is difficult for cell manufacture. A novel approach could solve the two problems at the same time (154). Banskota and colleagues invented engineered DNA-free virus-like particles (eVLPs) that efficiently package and deliver base editor or Cas9 ribonucleoproteins (154). These fourth-generation eVLPs are able to mediate efficient base editing in several primary mouse and human cell types, overcoming cargo packaging, release, and localization bottlenecks. This method offers high efficiency due to viral-vector based delivery and high precision due to its transient protein-based delivery. Nevertheless, this approach demonstrates a proof-of-concept (POC) power, yet waiting more proof of efficacy in the near future.

**Figure 6:**
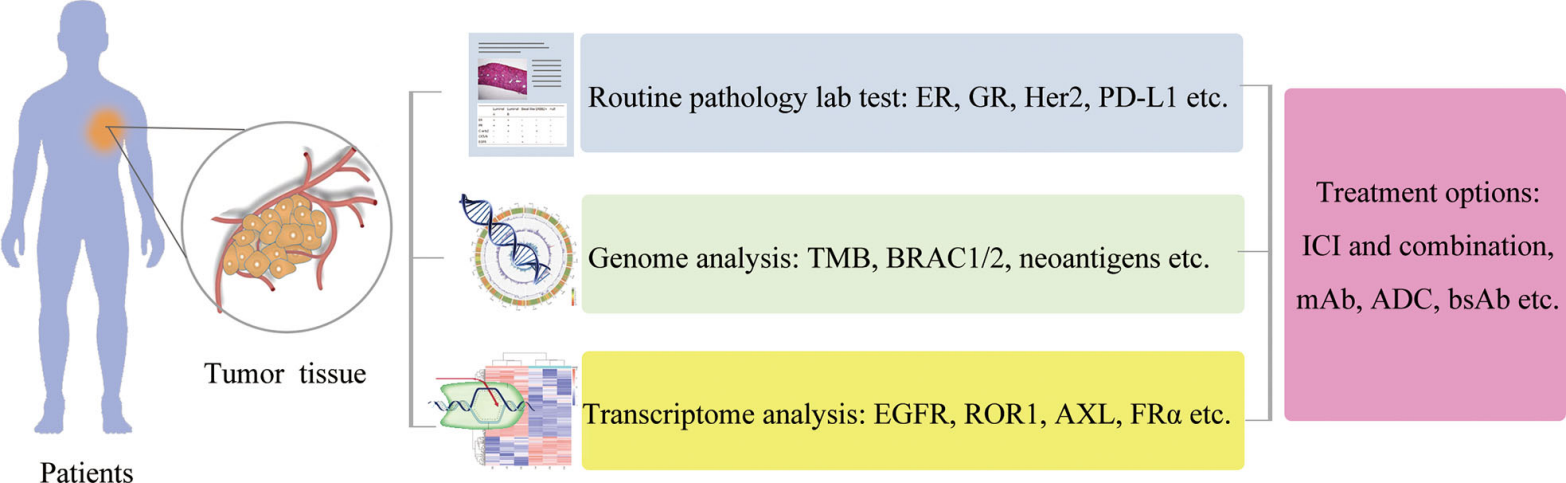
The integrated strategies for TNBC treatment.

**Figure 7:**
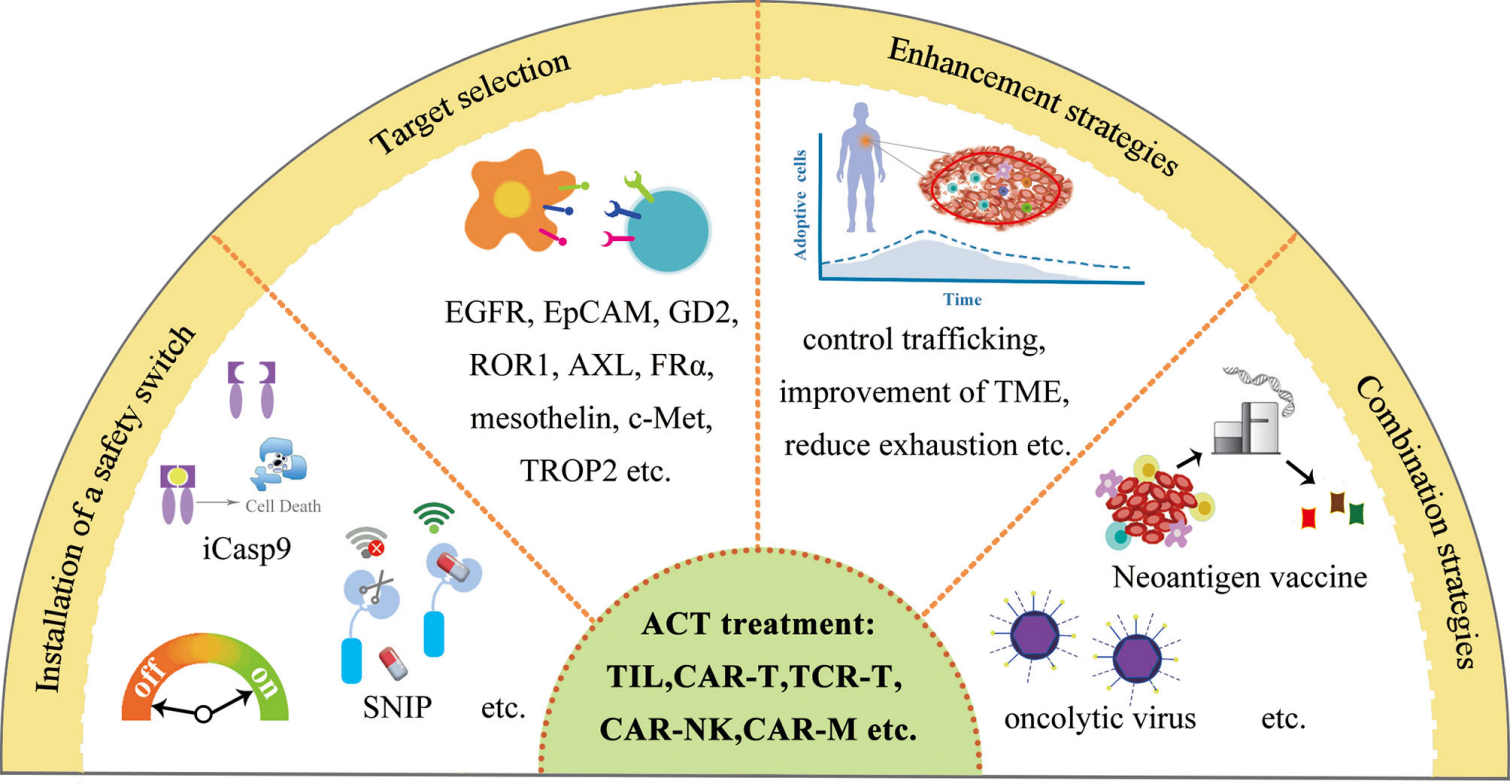
The improvements of ACT for TNBC treatment.

TNBC is a complex disease, and requires the integration strategies for TNBC treatment, with more comprehensive diagnoses utilizing genomic analysis and transcriptomic analysis at one end and combinatorial treatment strategies at the other end (**Figure 6**). In addition, the improvements of ACT for TNBC treatment are also required to overcome the current limitations in clinics, including many strategies such as the installation of a safety switch (155), target selection, immune cell function enhancement strategies (156), and the combinatorial strategies (**Figure 7**).

## Conclusions

TNBC is still the most intractable subtype of breast cancer, and the optimal treatment strategy for patients with TNBC remains a major unmet need. The molecular heterogenicity nature of TNBC requires the development of many treatment approaches with multiple targets. Novel effective therapeutic options, such as target therapy including PARP inhibitors; CDK inhibitors; immune molecule-based therapies including cytokines, mAbs, ADCs, bsAbs, ICIs, and neoantigen cancer vaccines; OV-based therapies; and ACT-based therapies including TIL, CAR-T, CAR-NK, CAR-M, and TCR-T are revolutionizing the therapeutic algorithm in both the preclinical-stage and clinical-stage settings. Along with chemotherapy, the new combinatorial therapeutic scenario has a full potential to improve the outcomes of patients with TNBC. Immunotherapy is still in the early developmental stage and has many challenges to overcome, requiring further explorations; both basic and translational research are warranted to lead to new opportunities in the treatment of TNBC and other cancers currently under a unmedicated condition in the future.

## Author Contributions

CYL drafted the manuscript. PPW, SQH, and JJZ designed the figure and tables and critically revised the manuscript. YYS and JXW conceived and critically revised the manuscript and tables. All authors contributed to the article and approved the submitted version.

## Funding

This work was supported by “Double First-Class” start-up funds from Beijing University of Chinese Medicine to JWX; No. 1000041510155.

## Conflict of Interest

The authors declare that the research was conducted in the absence of any commercial or financial relationships that could be construed as a potential conflict of interest.

## Publisher’s Note

All claims expressed in this article are solely those of the authors and do not necessarily represent those of their affiliated organizations, or those of the publisher, the editors and the reviewers. Any product that may be evaluated in this article, or claim that may be made by its manufacturer, is not guaranteed or endorsed by the publisher.
